# Identification and Validation of an m6A-Related LncRNA Signature to Predict Progression-Free Survival in Colorectal Cancer

**DOI:** 10.3389/pore.2022.1610536

**Published:** 2022-08-11

**Authors:** Yong Zhang, Lu Li, Feifei Chu, Xingguo Xiao, Li Zhang, Kunkun Li, Huili Wu

**Affiliations:** ^1^ Department of Gastroenterology, Zhengzhou Central Hospital Affiliated to Zhengzhou University, Zhengzhou, China; ^2^ Branch Center of Advanced Medical Research Center, Zhengzhou Central Hospital Affiliated to Zhengzhou University, Zhengzhou, China

**Keywords:** colorectal cancer, lncRNA, signature, m6A, progression free survival

## Abstract

The RNA methylation of N6 adenosine (m6A) plays a crucial role in various biological processes. Strong evidence reveals that the dysregulation of long non-coding RNAs (lncRNA) brings about the abnormality of downstream signaling in multiple ways, thus influencing tumor initiation and progression. Currently, it is essential to discover effective and succinct molecular biomarkers for predicting colorectal cancer (CRC) prognosis. However, the prognostic value of m6A-related lncRNAs for CRC remains unclear, especially for progression-free survival (PFS). Here, we screened 24 m6A-related lncRNAs in 622 CRC patients and identified five lncRNAs (SLCO4A1-AS1, MELTF-AS1, SH3PXD2A-AS1, H19 and PCAT6) associated with patient PFS. Compared to normal samples, their expression was up-regulated in CRC tumors from TCGA dataset, which was validated in 55 CRC patients from our in-house cohort. We established an m6A-Lnc signature for predicting patient PFS, which was an independent prognostic factor by classification analysis of clinicopathologic features. Moreover, the signature was validated in 1,077 patients from six independent datasets (GSE17538, GSE39582, GSE33113, GSE31595, GSE29621, and GSE17536), and it showed better performance than three known lncRNA signatures for predicting PFS. In summary, our study demonstrates that the m6A-Lnc signature is a promising biomarker for forecasting patient PFS in CRC.

## Introduction

As a common gastrointestinal cancer, colorectal cancer (CRC) has a high incidence and mortality rate [1]. According to the latest cancer statistics published in 2022, there are approximately 1.93 million new cases of CRC worldwide (10% of all new cancer cases); while about 0.94 million CRC cases resulted in the death of the patient (9.4% of all cancer fatalities)[2]. Though there has been good progress made in CRC therapy over the past 3 decades, patients with progressed or advanced CRC still have a high tendency towards relapse and metastasis in the following years and a poor prognosis, even after radical treatment [3]. For early detection of CRC incidence and risk assessment, many effective biomarkers have been developed and applied in clinic, such as carcinoembryonic antigen (CEA) [4]. However, due to the genomic and evolutionary heterogeneity of CRC, the clinical application of existing markers is not always effective. Therefore, it is necessary to uncover new molecular biomarkers to improve CRC prognosis, especially for progression-free survival (PFS).

In the human genome, over 90% of regions can generate transcripts, while 98% of these transcripts cannot encode proteins and are known as non-coding RNAs. Among those, long non-coding RNAs (lncRNA) have garnered extensive scientific attention because of their tissue-specific expression and universal regulatory functions [5]. Growing evidence reveals that lncRNAs can act as crucial regulators on multiple layers, such as dosage compensation effect, epigenetic regulation, and transcriptional and post-transcriptional regulation [6, 7]. Furthermore, lncRNAs are widely observed to be dysregulated in diverse cancer types including CRC, and many have been subject to experiments to demonstrate their contribution to tumor initiation and progression, metastasis, and even drug resistance [8, 9]. Previous studies revealed that some lncRNA signatures are relevant to survival outcome in CRC patients, suggesting the crucial role of lncRNA expression in predicting prognosis [10–12].

As the most common type of RNA modification, methylation of N6 adenosine (m6A) is recurrently reported to participate in both normal physiological processes and disease development [13]. The m6A modification is mainly mediated by three kinds of regulators, including RNA binding proteins (readers), methyltransferases (writers), and demethylases (erasers) [14]. The identification and investigation of m6A regulators has deepened the understanding of gene expression regulation on the post-transcriptional level [15]. Meanwhile, the dysregulation of these m6A regulators has been repeatedly observed to affect tumor cell biological phenotypes [16]. Notably, m6A regulators could also serve as single or combined biomarkers for cancer in clinical practice, such as predicting prognosis [17–19]. For example, a combined m6A marker (YTHDC2 and HNRNPC) can predict patients’ survival in head and neck cancer. Recently, most m6A regulators have been shown to affect lncRNA generation and action [20, 21], which has attracted extensive interest about modifications to cancer lncRNAs and their clinical application in precision oncology. At present, studies have been conducted on the interaction between lncRNAs and m6A regulators in multiple cancer types, and some potential clinical biomarkers have been identified for predicting patient survival [22–24].

However, there were few effective m6A-based biomarkers for predicting CRC survival, especially m6A-targeted lncRNAs. Thus, exploiting a prognostic biomarker based on m6A-related lncRNAs will be beneficial for guiding CRC practice. Considering that large studies have explored the value of the m6A-related lncRNA signature for predicting overall survival (OS) in CRC [25–27], we focused on PFS. In the current study, we developed and validated an m6A-Lnc signature to predict PFS in CRC patients.

## Materials and Methods

### Data Resource

Twenty protein-coding genes that functioned as m6A regulators[28] were collected, i.e., 11 readers (YTHDF2, YTHDF3, YTHDC1, YTHDC2, YTHDF1, RBMX, HNRNPC, HNRNPA2B1, IGF2BP1, IGF2BP2, and IGF2BP3), seven writers (METTL3, METTL14, RBM15, RBM15B, WTAP, VIRMA, and ZC3H13), and two erasers (ALKBH5 and FTO). We obtained RNA-Seq expression data (including FPKM and read count) and clinical data on 622 CRC (including colon cancer and rectal cancer) patients from the TCGA project (https://xenabrowser.net/datapages/). To validate the prognostic model, we additionally obtained six CRC datasets from the Gene Expression Omnibus (GEO), i.e., GSE17538 (210 patients), GSE39582 (557 patients), GSE33113 (89 patients), GSE31595 (33 patients), GSE29621 (53 patients), and GSE17536 (145 patients), totaling 1,077 CRC patients. They were from the GPL570 platform of U133 plus 2 arrays, which was suitable for probe annotation to obtain lncRNA expression. Gencode.v34 was used for lncRNA annotation.

### Identification of m6A Related LncRNAs

The differentially expressed lncRNAs were identified by comparing the expression profiles between tumor and normal samples. For the expression data (read count) detected by RNA-seq, we performed the differential expression analysis using R package DESeq2 [29] with FDR≤0.05 and fold change ≥2 or ≤1/2. In order to make the lncRNAs with enough expression and detectable by array, we only kept the differentially expressed lncRNAs with high expression (median FPKM>1) and with probe annotation for the GPL570 platform. Then, m6A-related lncRNAs were determined based on M6A2Target [30] and expression correlation by using four criteria as follows:1) lncRNAs were methylated or demethylated by m6A writers or erasers; 2) or lncRNAs were binding to m6A readers; 3) or the expression level of lncRNAs was influenced by over-expression or knock down of m6A regulators recorded in M6A2Target; 4) and lncRNAs were co-expressed with at least one m6A regulator in the TCGA CRC dataset (*p* value < 0.05 and Pearson’s coefficient >0.2 or < −0.2).

### Development of Prognostic m6A-Lnc Signature in CRC

For m6A-related lncRNAs, we utilized univariate Cox regression analysis to determine candidate factors for PFS. Based on the candidate lncRNAs, we performed LASSO analysis to get succinct and effective prognostic lncRNAs. LASSO analysis was implemented with functions cv.glmnet and glmnet in R package glmnet. The lncRNAs with LASSO regression coefficient not equal to 0 were retained. CRC patients were stratified based on lncRNA expression above or below the median. The survival curves were plotted using the Kaplan-Meier method, and the survival difference of two patient groups was estimated with the log-rank test (*p* value < 0.05).

The m6A-lncRNA signature model was established with a formula: m6A-LncScore = 0.32* SLCO4A1-AS1 expression +0.41* MELTF-AS1 expression +0.44* SH3PXD2A-AS1 expression +0.39*H19 expression +0.48* PCAT6 expression, where the figures before lncRNAs represent regression coefficients in univariate Cox regression analysis.

### Prognostic Evaluation Using m6A-Lnc Signature

CRC patients were stratified into two groups based on whether m6A-LncScore was above or below the median. Receiver operating characteristic (ROC) curve analysis and Area Under Curve of ROC (AUC) was utilized to show prediction power according to m6A-LncScore and other factors. Multi-variate Cox regression analysis was employed to determine the independent prognostic factors for PFS with adjustment for other potential clinicopathologic factors, i.e., age, gender, tumor stage, AJCC-T, AJCC-N, and AJCC-M. A nomogram and calibration plot were adopted to display the predictive ability and power of multiple features using R package rms. The model selection for the nomogram was performed by a backward step-down selection process using a threshold of *p* value < 0.05. Calibration curves were used to assess the calibration of the nomogram, accompanied by the Hosmer-Lemeshow test.

### Quantitative RT-PCR

Our in-house CRC cohort included 55 pairs of fresh specimens from CRC patients (tumor and matched adjacent normal tissue) without radiotherapy or chemotherapy, which were immediately stored in liquid nitrogen after surgery (Supplementary Table S1). All specimens were collected from Zhengzhou Central Hospital affiliated with Zhengzhou University between 2019 and 2020 and this study was approved by the Zhengzhou Central Hospital affiliated with Zhengzhou University. All subjects underwent rigorous screening and provided informed consent.

Quantitative RT-PCR (qRT-PCR) was employed to detect the RNA expression of the five lncRNAs (SLCO4A1-AS1, MELTF-AS1, SH3PXD2A-AS1, H19, and PCAT6). In brief, total RNAs of 55 pairs of tissue specimens were extracted using the Trizol method. After testing for concentration, purity, and integrity, an equal number of RNAs was used to synthesize cDNA. Finally, the SYBR Green Quantitative Kit (DBI, Germany) and 7500 Fast Quantitative PCR System (AB, United States) were used for detection. The housekeeping gene GAPDH was used as an internal reference, and the relative gene expression was expressed as 2^−ΔΔCt^. Primer sequences are shown in Supplementary Table S2.

## Results

### The m6A-Related LncRNAs in CRC

**FIGURE 1:**
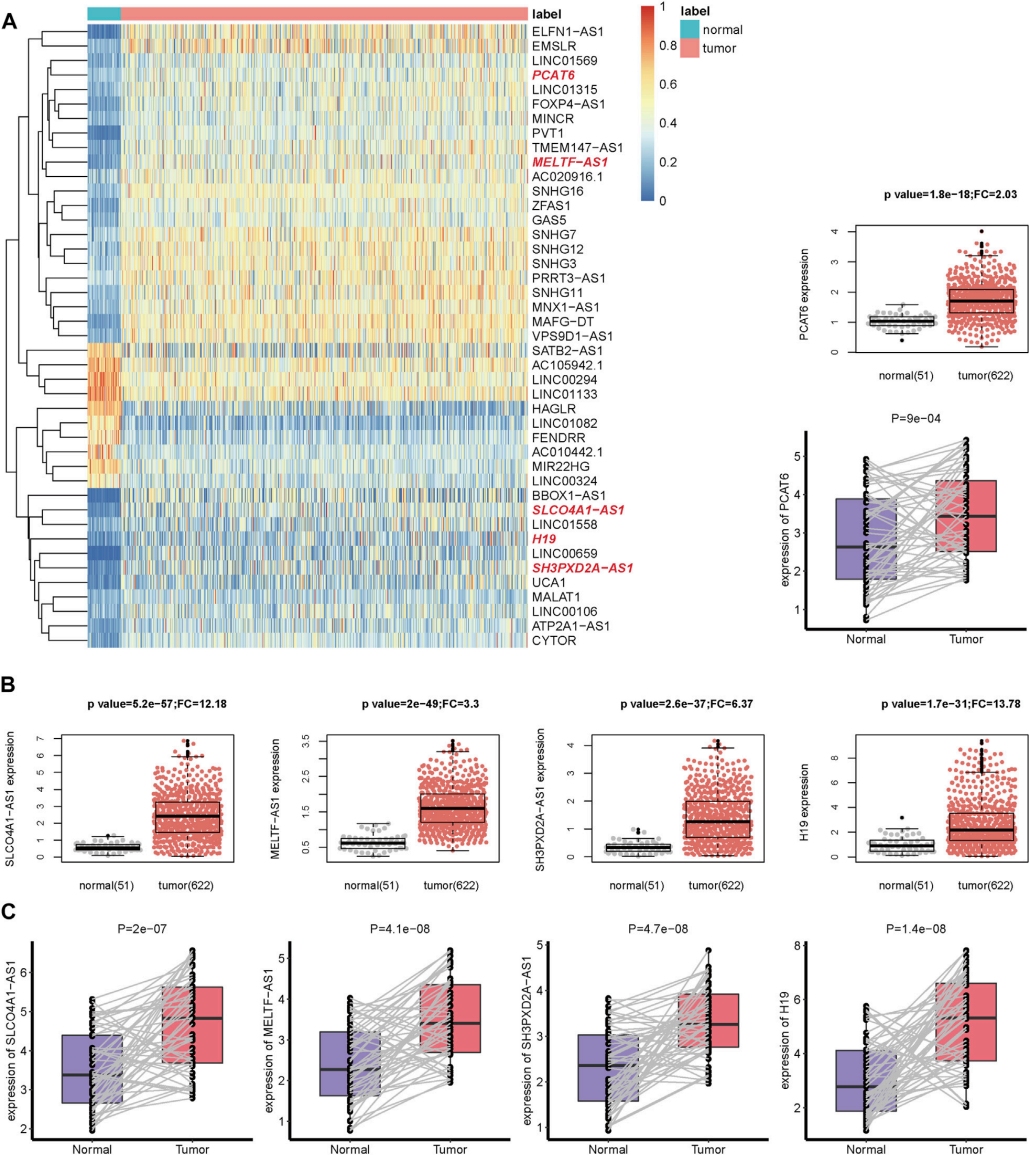
The gene expression of m6A-related lncRNAs in CRC. **(A)** The heatmap of 43 m6A-related lncRNAs expression in tumor and normal samples from TCGA dataset. The lncRNAs highlighted in red color in the heatmap are the lncRNAs in **(B,C)**. Complete hierarchical clustering based on euclidean distance was used. **(B)** The boxplot and beeswarm plot of five prognostic lncRNAs expression in tumor and normal samples from the TCGA dataset. **(C)** The boxplot of the five lncRNAs expression in 55 pairs of tissues (tumor and matched adjacent normal samples) from 55 CRC patients in our in-house cohort.

Compared with 51 normal adjacent samples, 3452 differentially expressed lncRNAs were identified in 622 CRC tumor samples, which comprised 2212 up-regulated and 1240 down-regulated lncRNAs (Supplementary Figure S1). Only 157 lncRNAs with high expression (median FPKM>1) remained. In order to enable lncRNAs to be verified by other datasets, we only focused on 43 recurrent lncRNAs (Figure 1A and Supplementary Table S3), whose expression could be also detected by the GPL570 platform. Interestingly, 18 of the 43 lncRNAs could interact with m6A regulators in NPInter V4 [31] (Supplementary Table S4); 32 of 43 lncRNAs and 41 of 43 lncRNAs could act as miRNA sponges and indirectly regulate m6A regulators via miRNAs in starBase V3 [32] and DIANA-LncBase V3 [33], respectively. For example, lncRNA PCAT6 bound to RBM15 and IGF2BP3 in several cancer cell lines, which were determined by CLIP and eCLIP technology.

Considering that these lncRNAs may act as the targeting genes of m6A modification, we further identified 24 m6A-related lncRNAs. They could receive m6A modification and bind to m6A readers, or their expression could be influenced by over-expression or knock down of m6A regulators in the M6A2Target database (Supplementary Tables S5–S7). Meanwhile, they were significantly co-expressed with at least one m6A regulator in the TCGA dataset (Supplementary Table S8). Notably, PCAT6 was significantly co-expressed with 12 m6A regulators (one positive and 11 negative relationships), suggesting its role in m6A RNA methylation (Supplementary Figure S2).

### The m6A-Lnc Signature for Predicting PFS in CRC

**FIGURE 2:**
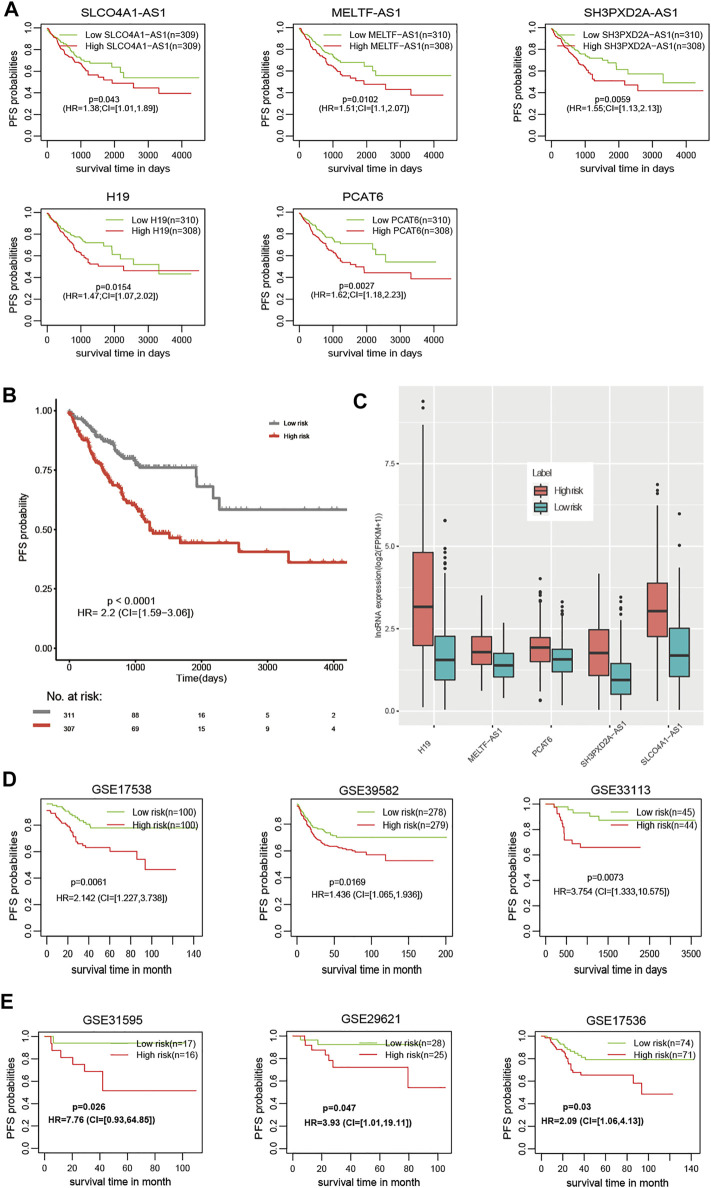
The prognostic value of the m6A-Lnc signature for predicting PFS in CRC. **(A)** The prognostic value of the five lncRNAs for predicting PFS. **(B)** Patients at high risk had significantly worse PFS than those at low risk in the TCGA dataset. **(C)** Patients at high risk had higher expression than those at low risk for the five lncRNAs. **(D)** The prognostic value of the m6A-Lnc signature for predicting PFS in GSE17538, GSE39582, and GSE33113. **(E)** The prognostic value of the m6A-Lnc signature for predicting PFS in GSE31595, GSE29621, and GSE17536.

For PFS, univariate Cox regression analysis identified five prognostic lncRNAs (SLCO4A1-AS1, MELTF-AS1, SH3PXD2A-AS1, H19, and PCAT6) (Table 1). LASSO analysis suggested they could form the simplest and most effective combination for predicting PFS (Supplementary Figure S3, Supplementary Table S9). Compared with normal samples, the five lncRNAs in CRC tumors had obviously higher expression (Figure 1B). We subsequently detected their expression status in our in-house CRC cohort by doing a qRT-PCR assay. Compared with matched adjacent normal tissues, their RNA expression was obviously up-regulated in most of the 55 tumors, and the difference was highly statistically significant (Figure 1C, Supplementary Table S10). The patients with high expression had a significantly shorter PFS time than other patients (Figure 2A).

**TABLE 1:** The univariate Cox regression analysis result of five lncRNAs for predicting PFS in the TCGA dataset.

LncRNA Name	Regression coefficient	HR	95% CI	P value
SLCO4A1-AS1	0.33	1.38	[1.1, 1.89]	0.044
MELTF-AS1	0.41	1.51	[1.1, 2.07]	0.011
SH3PXD2A-AS1	0.44	1.55	[1.13, 2.13]	0.006
H19	0.39	1.47	[1.07, 2.02]	0.016
PCAT6	0.48	1.62	[1.18, 2.23]	0.003

The m6A-Lnc signature was established with a formula: m6A-LncScore = 0.32* SLCO4A1-AS1 expression +0.41* MELTF-AS1 expression +0.44* SH3PXD2A-AS1 expression +0.39*H19 expression +0.48* PCAT6 expression. We calculated m6A-LncScore of 622 patients and divided the patients into two groups based on whether they scored above or below the median. Patients at high risk had a significantly shorter PFS time than those at low risk (Figure 2B). The high-risk patient group had higher lncRNA expression than the other group (Figure 2C). We also observed that m6A-LncScore was significantly co-expressed with 11 m6A regulators (two positive and nine negative relationships, Supplementary Figure S4).

Furthermore, we obtained the gene expression data of 1,077 CRC patients, including 210 patients from GSE17538, 557 patients from GSE39582, 89 patients from GSE33113, 33 patients from GSE31595, 53 patients GSE29621, and 145 patients from GSE17536, and validated the prognostic model in these six independent datasets (Figures 2D,E). Using the lower or upper quartile as the threshold, we also observed the statistical significance in most datasets (Supplementary Figures S5, S6), suggesting the robustness of the m6A-Lnc signature using different thresholds to classify patients as high or low risk. In addition, we found that the m6A-Lnc signature was also suitable for predicting overall survival (OS) in the TCGA CRC and COAD datasets (Supplementary Figure S7).

### The Prognostic Value of m6A-LncScore Was Independent of Clinicopathological Factors for PFS

To estimate whether m6A-LncScore could act as an independent factor in CRC to predict PFS, we performed univariate and multivariate Cox regression analysis. The results showed that m6A-LncScore (*p* < 0.0001; HR = 1.43, CI = 1.26–1.62) and several clinicopathological factors (tumor stage, AJCC‐T, AJCC‐N, AJCC‐M and CEA level) were significantly relevant to patient PFS in the TCGA dataset (Table 2). Considering that the information on tumor stage was relatively complete in the TCGA dataset and the other three datasets, while other factors in many patients were missing or even unrecorded, we only adopted tumor stage into the multivariate Cox regression analysis. After adjustment by tumor stage, m6A-LncScore was still significant (*p* = 0.0021; HR = 1.25, CI = 1.08–1.44) (Table 3), indicating its independent prognostic potential. The independent prognostic value of m6A-LncScore was validated in three other datasets (GSE17538, GSE39582, and GSE33133) (Supplementary Tables S11, S12).

**TABLE 2:** The univariate Cox regression analysis result of m6A-LncScore and clinicopathologic features for predicting PFS in the TCGA dataset.

Factors	Description	HR	95% CI	P value
m6A-LncScore (ref = Low)	m6A-LncScore = High	1.43	[1.26, 1.62]	<0.0001
Age	Age	1	[0.99, 1.01]	0.91
gender (ref = FEMALE)	Gender = MALE	1.26	[0.92, 1.74]	0.15
Stage (ref = I)	Stage = II	2.41	[1.14, 5.11]	0.02
	Stage = III	3.54	[1.67, 7.51]	0.001
	Stage = IV	13.42	[6.37, 28.29]	<0.0001
pT (ref = T1)	pT = T2	0.94	[0.2, 4.37]	0.93
	pT = T3	2.91	[0.72, 11.77]	0.13
	pT = T4	8.85	[2.12, 36.99]	0.0028
pN (ref = N0)	pN = N1	1.69	[1.13, 2.51]	0.0101
	pN = N2	4.21	[2.93, 6.06]	<0.0001
pM(ref = M0)	pM = M1 (ref = M0)	5.43	[3.82, 7.72]	<0.0001
Lymph node count (ref <19)	Lymph node count≥19	1	[0.98, 1.01]	0.35
CEA	CEA	1.0004	[1.0002, 1.0006]	<0.0001

**TABLE 3:** The multi-variate Cox regression analysis result of m6A-LncScore and clinicopathologic features for predicting PFS in the TCGA dataset.

Factors	Description	HR	95% CI	P value
m6A-LncScore (ref = Low)	m6A-LncScore = High	1.25	[1.08, 1.44]	0.0021
Stage (ref = I)	Stage = II	1.29	[0.29, 5.76]	0.74
	Stage = III	1.79	[0.38, 8.35]	0.46
	Stage = IV	6.15	[1.38, 27.43]	0.0173
pT (ref = T1)	pT = T2	0.77	[0.16, 3.78]	0.75
	pT = T3	1.31	[0.18, 9.58]	0.79
	pT = T4	2.5	[0.33, 18.84]	0.37
pN (ref = N0)	pN = N1	0.62	[0.24, 1.56]	0.31
	pN = N2	1.18	[0.47, 2.97]	0.72
^a^pM(ref = M0)	pM = M1	NA	NA	NA

a Since the M1 of AJCC‐pM completely equals to the stage IV tumor, and the information on AJCC‐pM is included in tumor stage, thus there will appear NA in pM when they were simultaneously added to Cox regression analysis. The result suggested that tumor stage and AJCC-pM are strongly correlated for predicting CRC prognosis.

**FIGURE 3:**
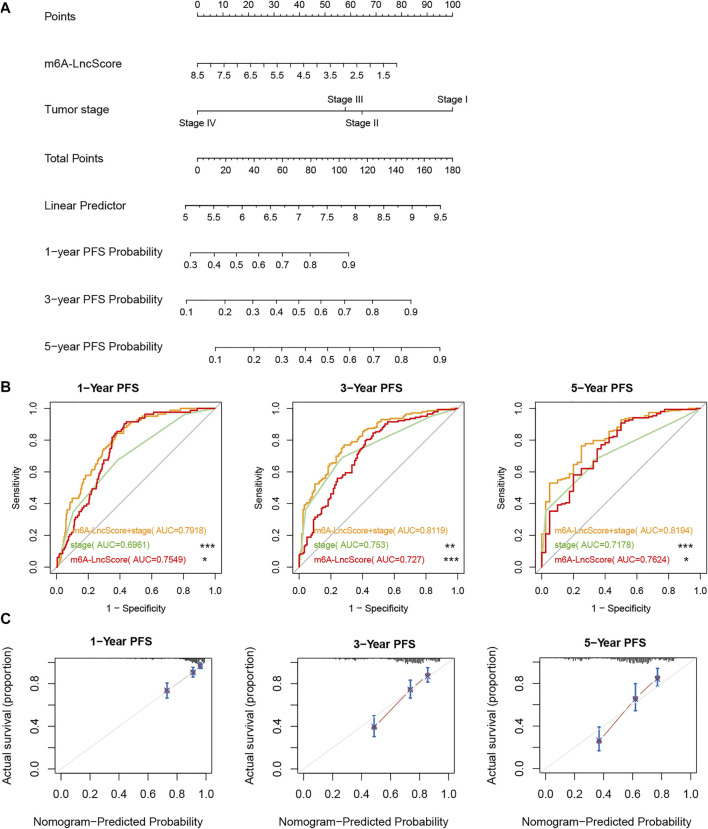
The accuracy of m6A-LncScore in predicting PFS considering clinicopathological factors. **(A)** The nomogram plot of m6A-LncScore for predicting PFS. **(B)** The ROC curve plot of m6A-LncScore for predicting PFS compared to tumor stage. The *p* value of AUC between the integrated model with m6A-LncScore and tumor stage was labeled, ***p < 0.005; **p < 0.01; *p < 0.05; ns *p* > 0.05. **(C)** The calibration plot of the model integrating m6A-LncScore with tumor grade for predicting PFS.

The result of nomogram analysis also showed the good predictive ability of m6A-LncScore, as well as clinicopathological factors (Figure 3A). Since tumor stage contained the complete information on AJCC‐T, AJCC‐N, AJCC‐M, the integrated model combining m6A-LncScore with independent prognostic factors (tumor stage and risk score) was further established. We found that the AUC of m6A-LncScore was 0.75, 0.73, 0.76 (for 1‐, 3‐, and 5‐year PFS, respectively), the AUC of tumor stage was 0.7, 0.75, 0.72, while the AUC of the model integrating m6A-LncScore with tumor stage was 0.79, 0.81, 0.82. The results suggested that the integrated model for predicting PFS was superior to m6A-LncScore or tumor stage (Figure 3B, Supplementary Tables S13, S14).

Furthermore, the calibration plot showed good consistency between observation and predictive values for 1‐, 3‐, and 5‐year PFS (Figure 3C). The ROC analysis in GSE17538, GSE39582, and GSE33113, also confirmed that m6A-LncScore had high accuracy in predicting patient PFS (Supplementary Figures S8–S10). In the TCGA dataset, even considering CEA level, we found that the integrated model for predicting PFS was superior to m6A-LncScore or CEA level (Supplementary Figure S11, Supplementary Table S15).

**FIGURE 4:**
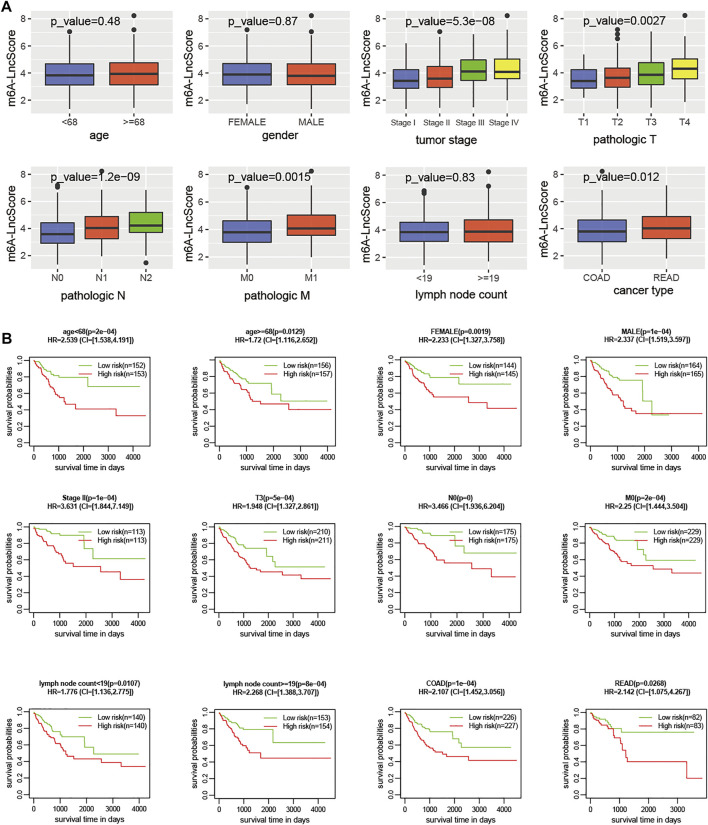
The correlation of clinicopathologic features with m6A-LncScore. **(A)** The m6A-LncScore was associated with clinicopathologic features. **(B)** Stratification analysis shows m6A-LncScore is not dependent on clinicopathologic features for predicting PFS.

We found some clinicopathological factors had a significant association with m6A-LncScore, especially tumor stage, AJCC‐T, AJCC‐N, and AJCC‐M (Figure 4A). When the patients were stratified by these factors, m6A-LncScore was still statistically significant for patients when comparing the high- and low-risk groups (Figure 4B). The results demonstrated that m6A-LncScore was completely independent of four factors (age, gender, lymph node count, and cancer type) and partially independent of another four factors (tumor stage II, AJCC T3, N0, and M0). Taken together, m6A-LncScore was an independent prognostic biomarker for PFS.

### The m6A-Lnc Signature Was Superior to Known LncRNA-Related Signatures

**FIGURE 5:**
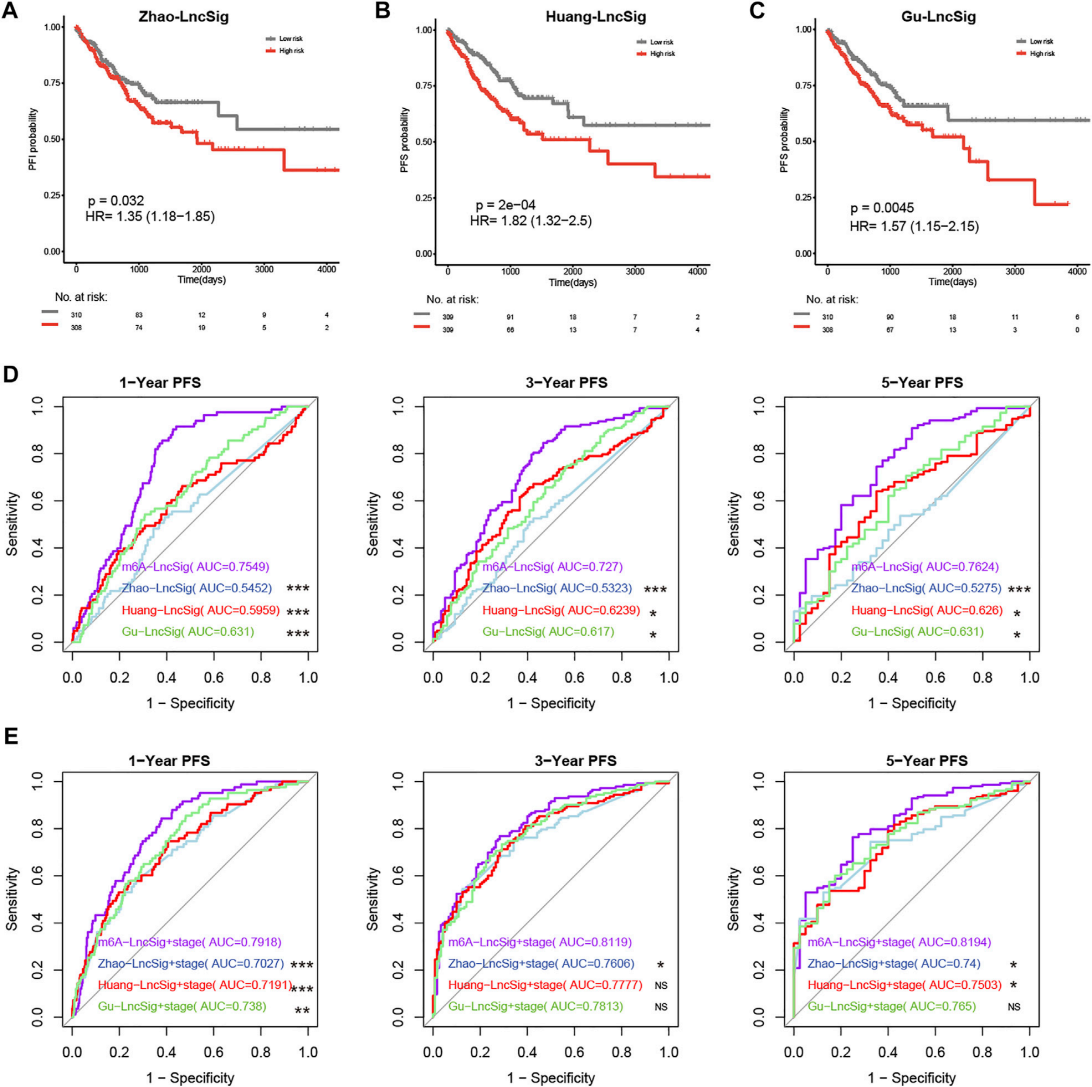
Comparison of predictive power for PFS across the m6A-Lnc signature and three known lncRNA signatures. **(A–C)** The prognostic value of three known signatures. **(D)** The predictive power of m6A-LncSig was significantly higher than other three signatures. **(E)** The predictive power of m6A-LncSig was comparable with or significantly higher than three other signatures when integrated with tumor grade. The *p* value of AUC between m6A-LncSig and three other lncRNA signatures was labeled, ***p < 0.005; **p < 0.01; *p < 0.05; ns *p* > 0.05.

Previous studies revealed three lncRNA-related signatures relevant to PFS in CRC patients [10–12]. Thus, we compared the prognostic potential of m6A-Lnc signature (called m6A-LncSig) to these three lncRNA-related signatures (Zhao-LncSig, Huang-LncSig, and Gu-LncSig). In TCGA patients, the three signatures had a similar tendency for high-risk patients to have a shorter PFS period. They all showed a good ability of predicting PFS (*p* = 0.032, HR = 1.35; p = 2e-04, HR = 1.82; *p* = 0.0045, HR = 1.57, log-rank test) (Figures 5A–C). Dependent ROC analysis was conducted to compare the prognostic power of m6A-LncSig and the three signatures in the TCGA dataset. In general, the AUC at 1‐, 3‐, and 5‐ PFS for the m6A-LncSig was 0.75, 0.73, and 0.76, which was significantly higher than that of Zhao-LncSig (AUC = 0.55, 0.53, 0.53), Huang-LncSig (AUC = 0.6, 0.62, 0.63), and Gu-LncSig (AUC = 0.63, 0.62, 0.63) (Figure 5D, Supplementary Tables S16). Even integrated with tumor stage, the predictive power (1‐year, 3‐year, and 5‐year PFS) of m6A-LncSig was comparable with or significantly higher than the other three signatures (Figure 5E, Supplementary Tables S16). These results demonstrated that the prognostic power of the m6A-lncRNA signature was superior to three known lncRNA-related signatures.

### The Biological Functions Associated With m6A-Lnc Signature

**FIGURE 6:**
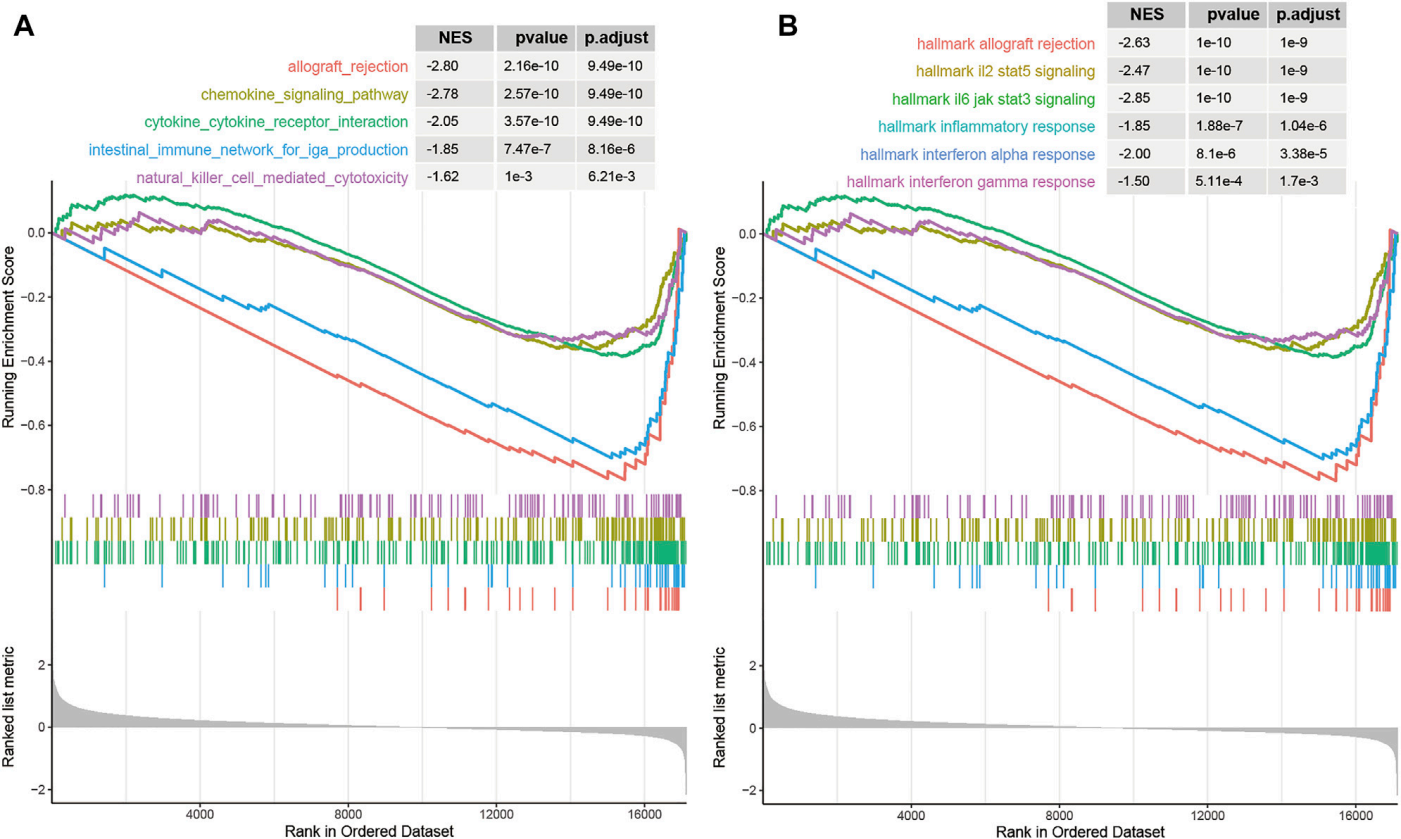
The biological functions associated with the prognostic signature. **(A)** immune-related pathways in KEGG. **(B)** immune-related cancer hallmark pathways.

Functional annotation was further conducted using gene-set enrichment analysis (GSEA) [34] for cancer hallmarks from MsigDB [35], which was implemented by R package “clusterProfiler” [36]. The differentially expressed genes between the high- and low-risk groups based on m6A-LncScore were enriched in immune-related cancer pathways and hallmarks (Figures 6A,B), such as interferon alpha/gamma response and inflammatory responses. The result indicated that the CRC patients with high m6A-LncScore had poor PFS time, which may be related to the immunosuppression of the tumor microenvironment.

## Discussion

As a new post-transcriptional modification, m6A can be installed by methyltransferases (i.e., writers) and removed by demethylases (i.e., erasers). It alters target-gene expression through a class of proteins (i.e., readers) recognizing and binding to methylated or demethylated RNA sequence, thus influencing biological processes and functions [37]. In mechanism, m6A is involved in multiple steps of RNA metabolism, such as RNA translation, degradation, alternative splicing, nucleo-cytoplasmic transport, and structural formation[38]. Growing evidence shows that m6A plays a dual role in cancer. On the one hand, the effect of m6A on cancer can be reflected in the change of m6A modification in tumor-related genes, thus influence tumor initialization and progression. On the other hand, the expression and activity of m6A regulator can be modulated, thereby influence m6A’s modification and interaction with target genes in tumor initialization and progression [39]. Many studies on m6A regulators have brought new insights to account for aberrant expression and the underlying mechanism in cancer. Thus, systematic investigation of these issues (such as m6A modification profile, post-modification regulation, and m6A regulators’ interaction with target genes), will contribute to revealing the mechanism of m6A in cancer and developing a potential therapeutic strategy [40].

Except for mRNA, non-coding RNAs (such as miRNAs, lncRNAs and circular RNAs) can also regulate m6A, or be regulated by m6A [41]. LncRNAs are also shown to be extensively m6A-modified, and carry out various functions such as the lncRNA-mediated ceRNA model, and XIST-mediated gene silencing [42]. The mutual regulation relationship between m6A methylation and lncRNAs can be seen in a normal intestinal epithelium cell as well as a CRC cell. For instance, m6A methylation could promote transcriptional repression *via* lncRNA XIST mediation in embryonic stem cells [43, 44]. LncRNA RP11’s upregulation, which is induced by the abnormality of m6A methylation, can promote the migration, invasion, and metastasis of CRC cells by positive upregulation of Zeb1 [45]. The m6A regulators play a crucial roles in achieving m6A methylation. Many studies have demonstrated that the dysregulation of m6A regulators can impact the generation and action of lncRNAs in cancer [13]. ALKBH5 promotes colon cancer progression by decreasing methylation of the NEAT1 [46]. Meanwhile, lncRNAs can also regulate m6A regulators to facilitate or suppress cancer progression. For example, LINC00470 inhibits the PTEN stability by binding to METTL3 and promotes gastric cancer progression [47]. LncRNA LINRIS stabilizes IGF2BP2 through the autophagy-lysosome pathway, and promotes MYC-mediated glycolysis to affect CRC cell growth [48]. Thus, investigation of the regulation relationship between m6A methylation and lncRNAs could provide new insights into the molecular mechanisms of cancer.

Several studies have revealed m6A regulators or their combinations were associated with cancer patient outcome [18, 49, 50]. Li et al. globally characterized the molecular landscape and clinical relevance of m6A regulators in 33 cancer types [28]. Recently, the m6A regulator signature (YTHDC2 and ALKBH5) [18] and m6A regulators (YTHDC2 and IGF2BP3) signature [51] were shown to have good predictive performance for OS in CRC. Some studies have explored the ability of m6A-related lncRNAs to predict survival in cancer patients [52–55]. However, none have succeeded in validating the signatures’ prognostic value in more than two additional datasets, which did not guarantee the robustness and extensibility of signatures. Furthermore, there have been no studies integrating m6A regulators and lncRNAs to predict PFS, which inspired us to investigate the prognostic value of m6A-related lncRNAs in CRC. Thus, we developed and validated an m6A-based lncRNA signature for predicting CRC PFS.

In our study, we observed that the differentially expressed genes between the high- and low-risk groups based on m6A-LncScore were enriched in immune-related cancer pathways and hallmarks (Figures 6A,B), such as interferon alpha/gamma responses, and inflammatory responses. As a key layer to mediate anti-inflammation and anti-tumor immunity [56], m6A regulator in malignant tumors is of great significance to understand the immune modulating function and develop new immunotherapeutic strategies [57]. For example, Han et al. emphasized that combining an immune checkpoint blockade with a YTHDF1 deficiency may bring extra benefits to patients with low response [58]. M6A modifications can cause changes in inflammation-related genes during inflammation. Large studies of m6A modified cross-linking, substrate genes, and modified regulation illustrated the mechanism of m6A action in inflammation [59]. For example, silencing m6A “reader” YTHDF2 increases the expression of MAP2K4 and MAP4K4 mRNA by stabilizing mRNA transcription, which activates MAPK and NF-κB signaling pathways, further inducing the expression of pro-inflammatory cytokines, and exacerbating the inflammatory response of LPS-stimulated macrophage 264.7 cells [60]. In the immune system, especially in tumor immunity, RNA methylation affects the maturation and response function of immune cells. Some recent studies have confirmed that RNA methylation can regulate tumor immunity, which also provides new ideas for the treatment of immune diseases and tumor immunotherapy in the future [61]. One study proved that RNA methylation played an essential role in maintaining T cell homeostasis [62]. The absence of METTL3 makes T cells stay in the naive T cell stage for longer via METTL3-mediated m6A methylation targeting the IL-7/STAT5/SOCS pathway.

In addition, some clinicopathological factors were significantly associated with m6A-LncScore, so stratification analysis was performed. M6A-LncScore was predictive for PFS completely independently of age, gender, lymph node count, cancer type, and CEA level (Figure 4B, Supplementary Figure S11), while it was partly independent of tumor stage, AJCC-T, AJCC-N, and AJCC-M. In summary, m6A-LncScore was more suitable for predicting PFS in patients with early-stage cancer, T3, N0, and M0 (Figure 4B, Supplementary Figure S13), which suggests its role in classification prediction and precision oncology.

Although we have revealed that m6A-LncScore was a risk factor for predicting CRC PFS, there are still some limitations to this study. First, the potential mechanisms of m6A-LncScore on functional phenotypes of CRC need further investigation through functional experiments. Second, more CRC specimens should be collected to verify the expression status of the five lncRNAs. Finally, there is a lack of follow-up data in our cohort to further validate the prognostic value of m6A-LncScore.

In this study, we developed an m6A-based lncRNA signature predicting PFS in 622 CRC patients, and validated it in another 1,077 patients from six GEO datasets. It was shown to be an independent prognostic factor and superior to three existing lncRNA signatures. In sum, the m6A-Lnc signature could serve as a potential prognostic biomarker, which might benefit our understanding of m6A modification of lncRNAs, and guide the individualized treatment of CRC patients.

## Data Availability

The datasets presented in this study can be found in online repositories. The names of the repository/repositories and accession number(s) can be found in the article/Supplementary Material.
