# Supplementary material for: Identification and Validation of an m6A-Related LncRNA Signature to Predict Progression-Free Survival in Colorectal Cancer
Source: Pathol Oncol Res. 2022 Aug 11;28:1610536. doi: 10.3389/pore.2022.1610536 (PMC9407446; doi:10.3389/pore.2022.1610536)
Supplement: Supplementary file 1 [file DataSheet1.DOCX]

**Supplemental Information：**

**Legends of Supplementary Figures**


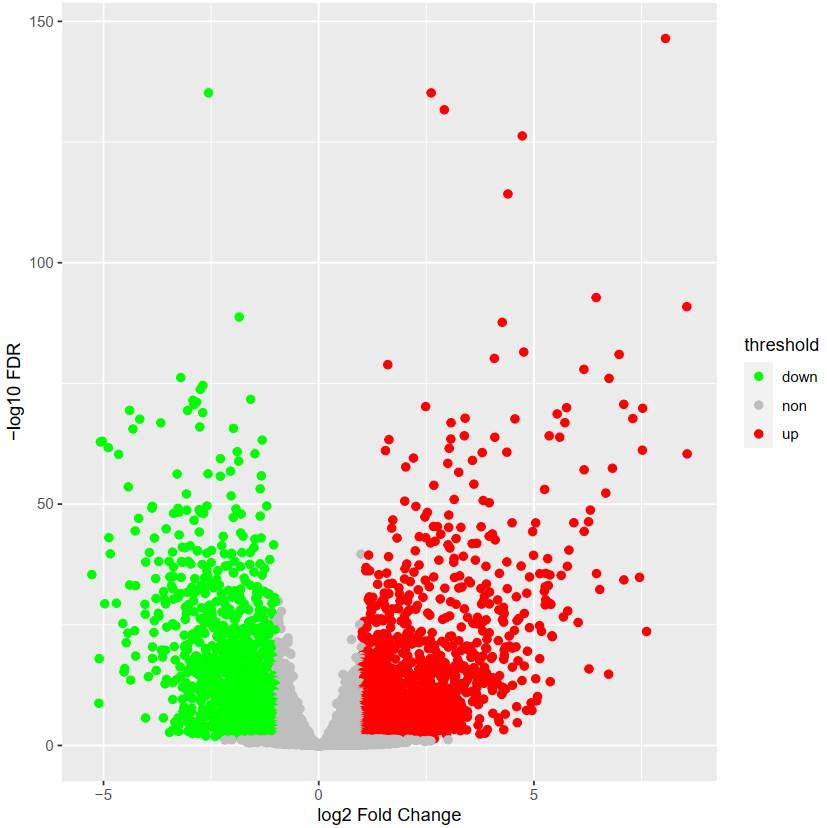


**Figure S1** Volcano Plot of differentially expressed lncRNAs in TCGA CRC datasets.


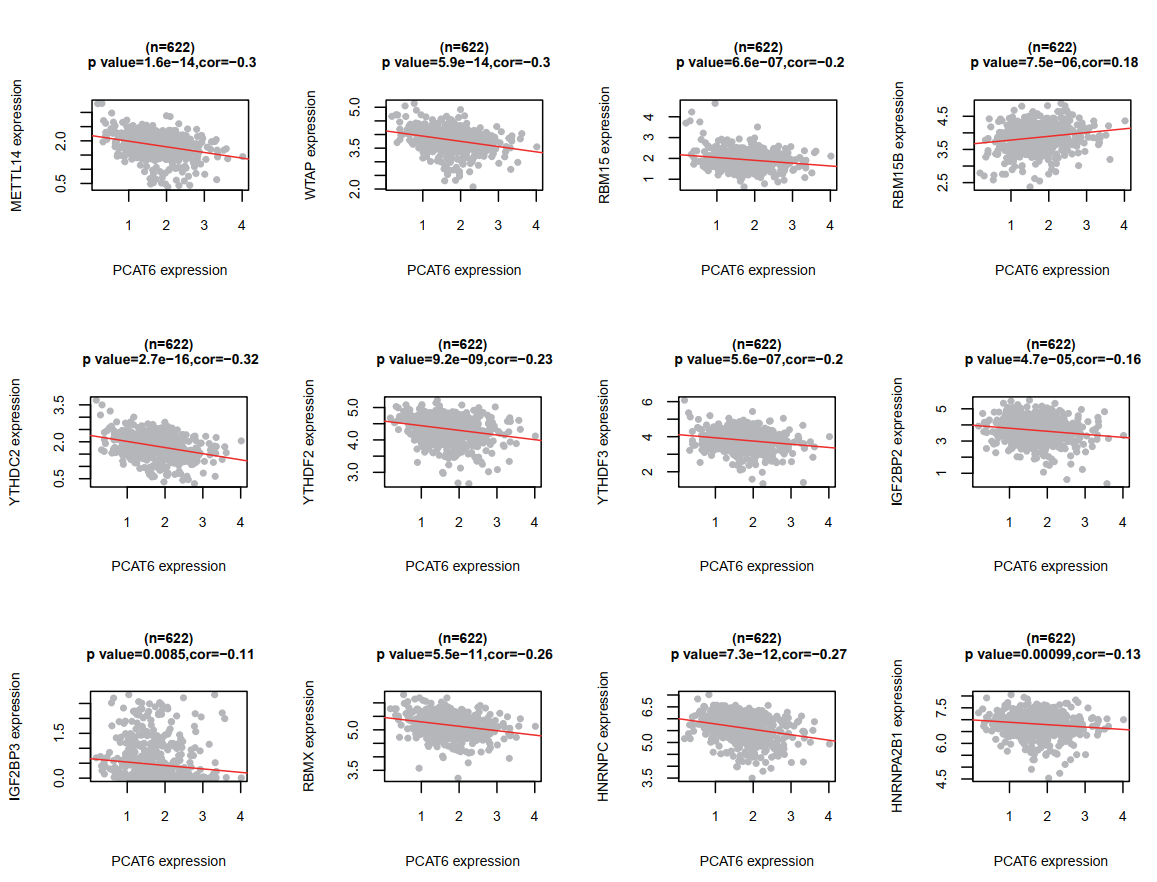


**Figure S2** PCAT6 is significantly co-expressed with 12 m6A regulators in TCGA dataset.


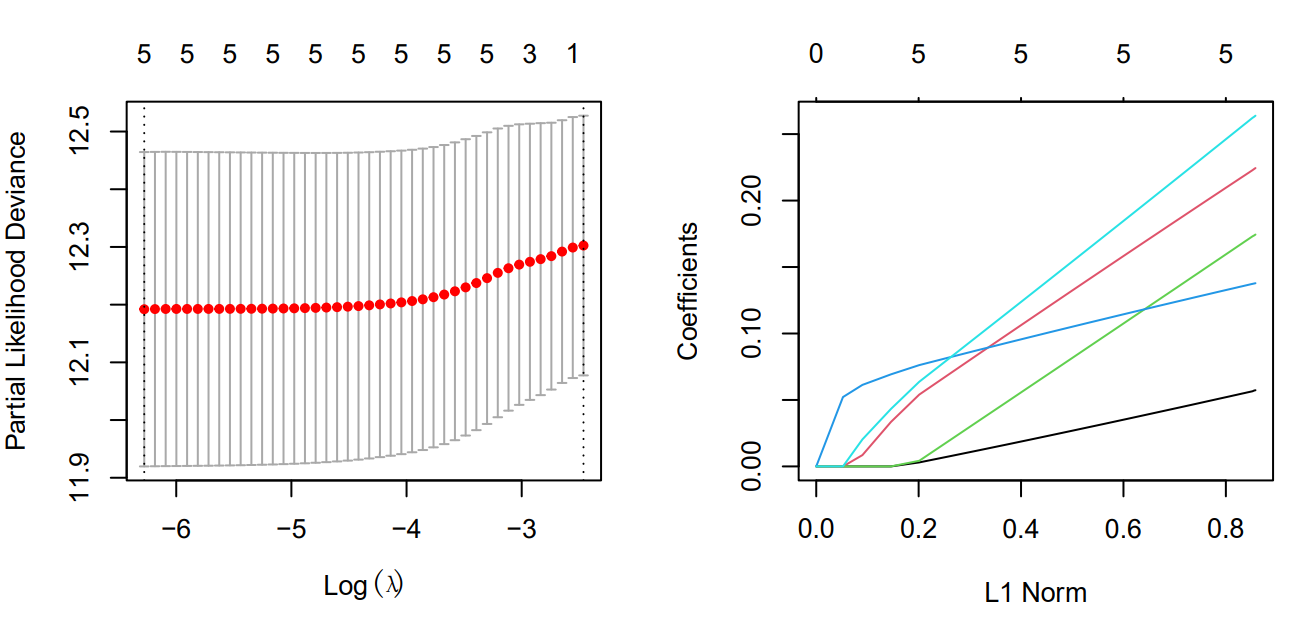


**Figure S3** LASSO analysis result of 5 lncRNAs.


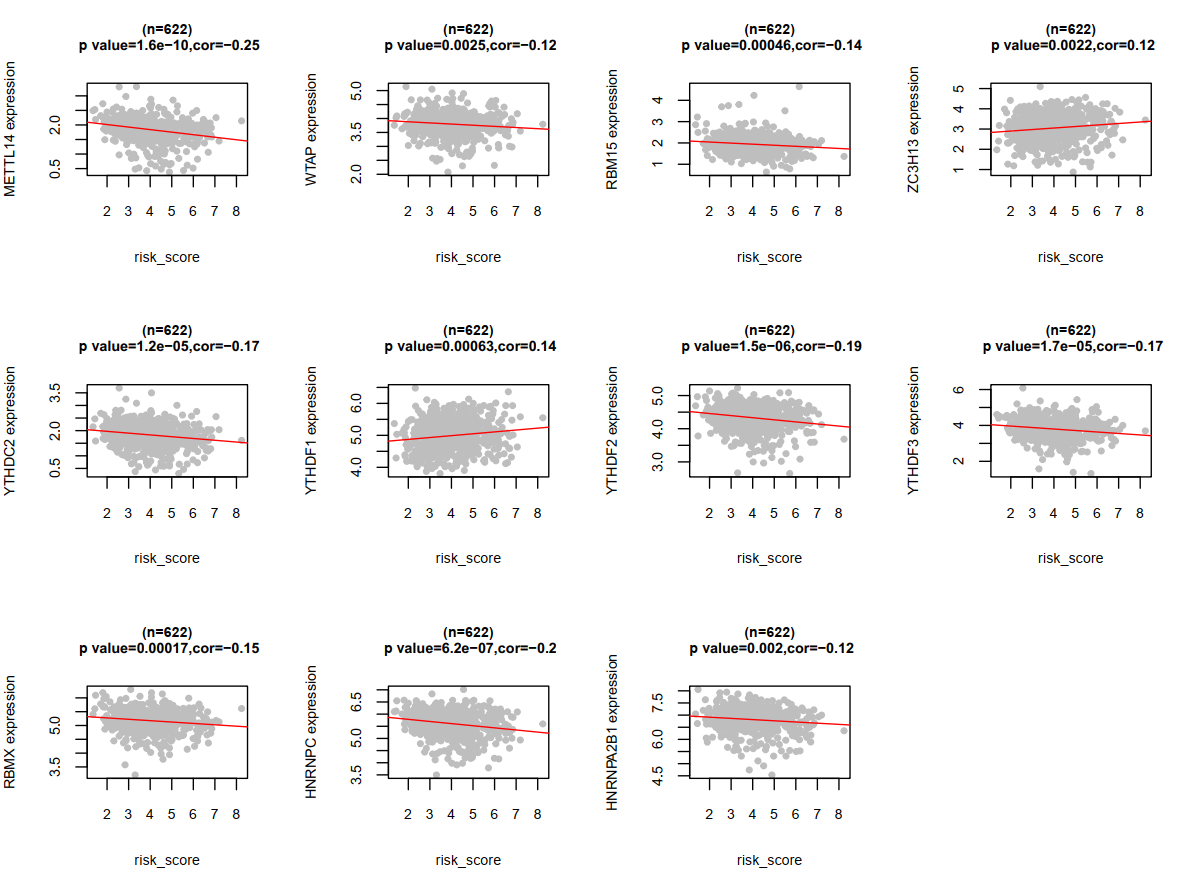


**Figure S4** The m6A-LncScore is significantly co-expressed with 11 m6A regulators in TCGA dataset..


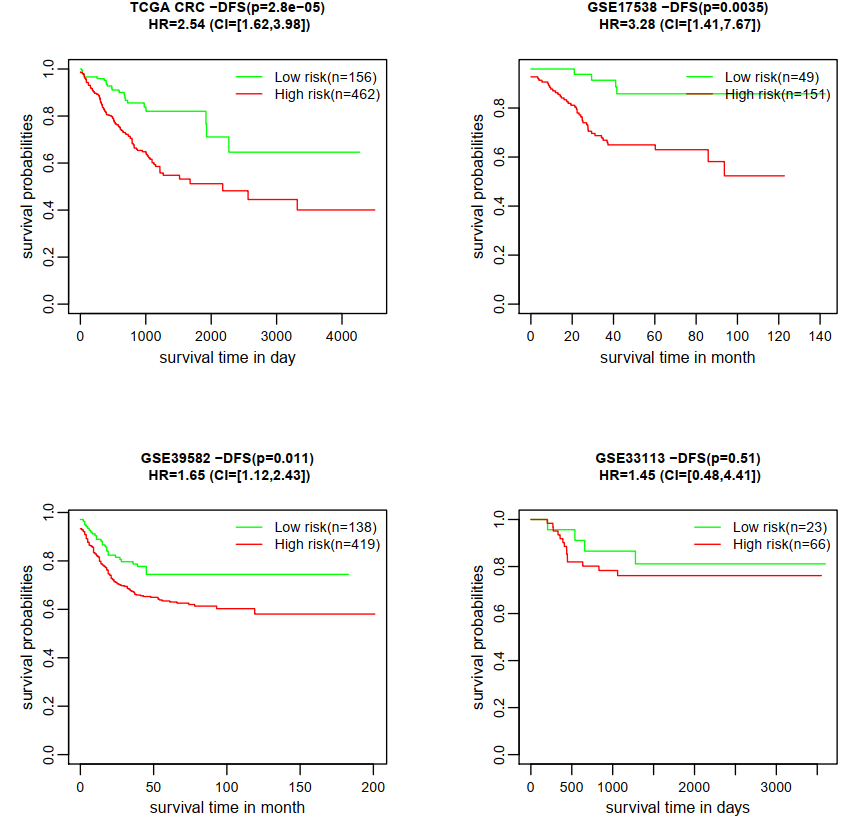


**Figure S5** Patients with high risk (lower quartile as the threshold) had significantly worse PFS than those with low risk in TCGA, GSE17538 and GSE39582. In GSE33113, patients with high risk had shorter PFS time than those with low risk, but p value of the difference was not significant (p value=0.51).


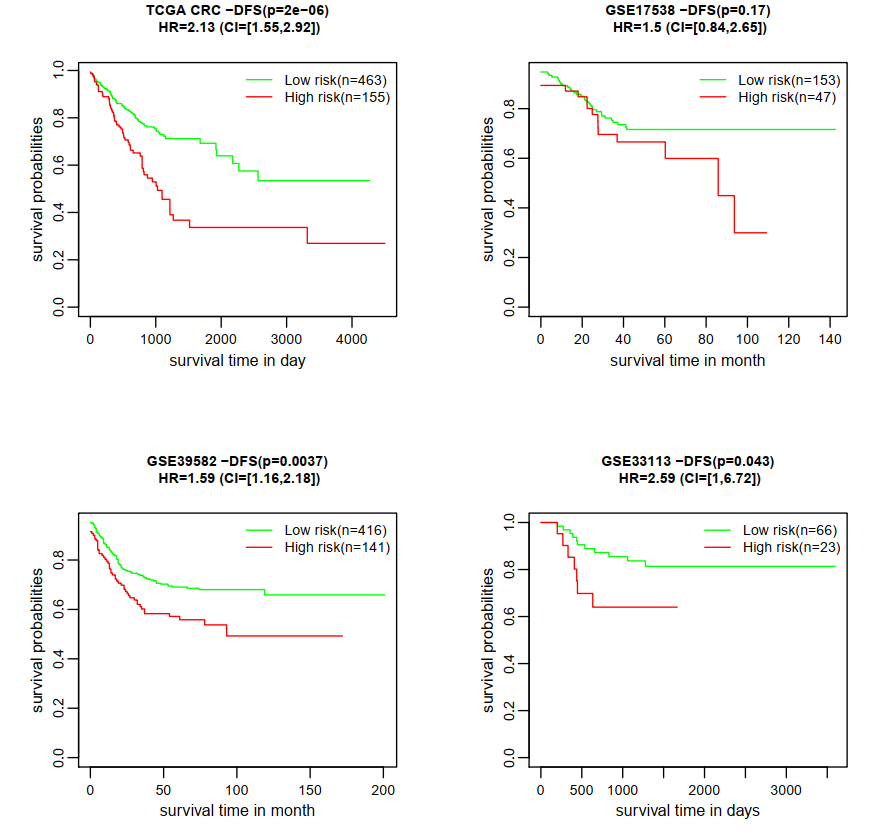


**Figure S6** Patients with high risk (upper quartile as the threshold) had significantly worse PFS than those with low risk in TCGA, GSE39582 and GSE33113. In GSE17538, patients with high risk had shorter PFS time than those with low risk, but p value of the difference was not significant (p value=0.17).


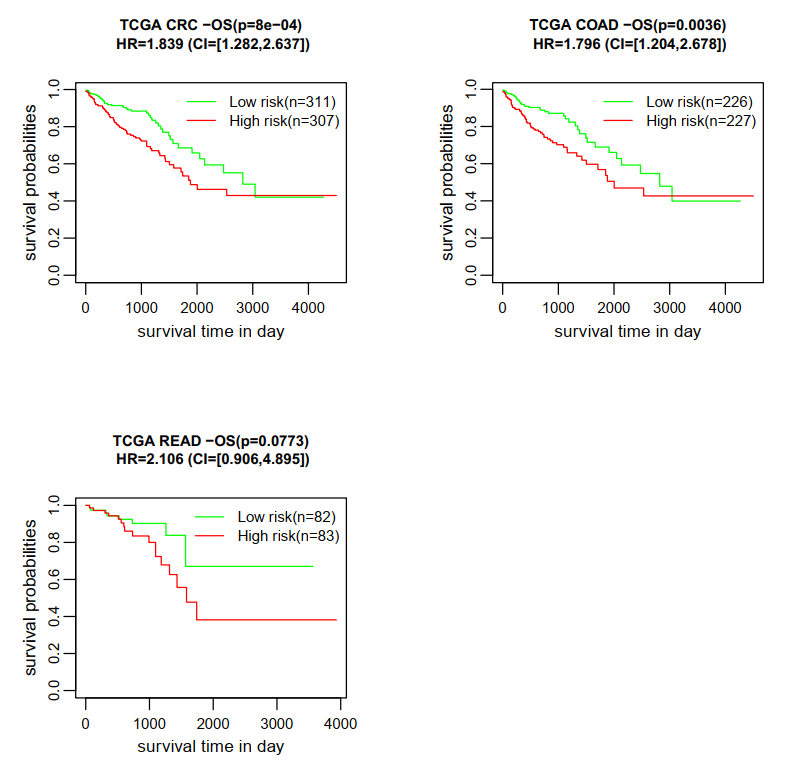


**Figure S7** The m6A-Lnc signature is suitable for predicting overall survival in TCGA CRC and COAD datasets. In READ dataset, there is a tendency, but p value of the difference was not significant (p value=0.073).


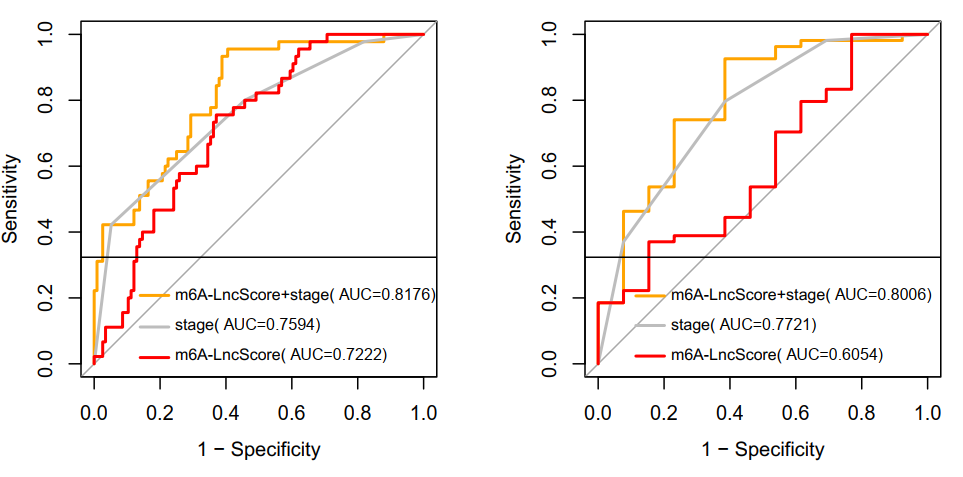


**Figure S8** The ROC curve plot of m6A-LncScore for predicting PFS compared to tumor stage in GSE17538. Since the data did not have a long enough follow-up time, only 1-year and 3-year PFS were predicted, not 5-year PFS.


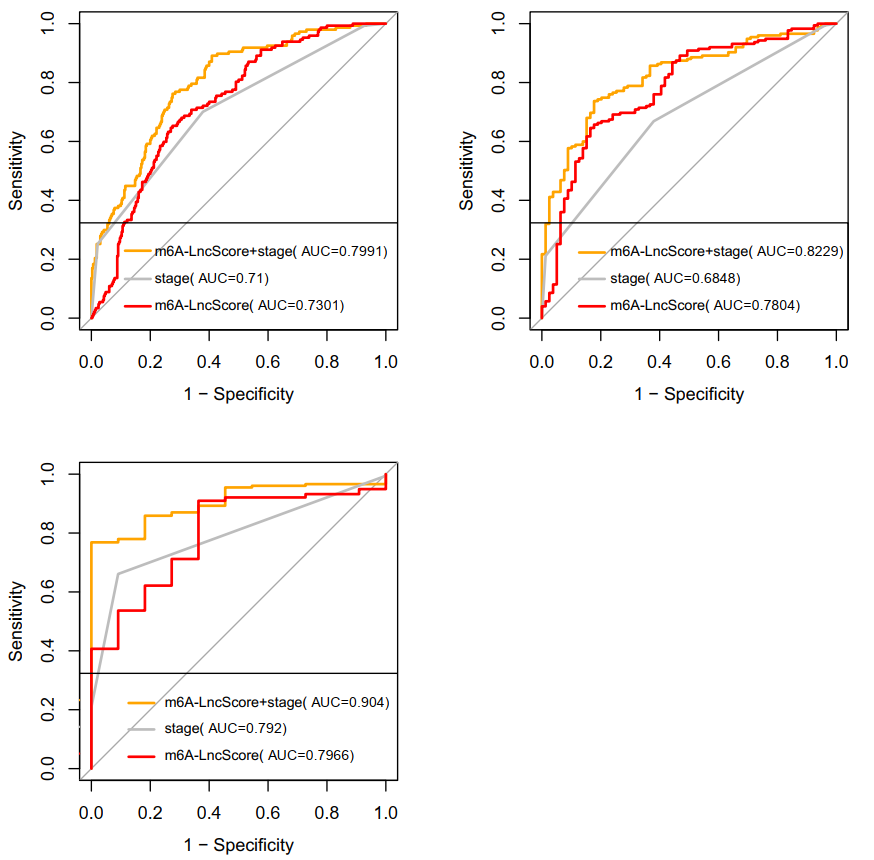


**Figure S9** The ROC curve plot of m6A-LncScore for predicting PFS compared to tumor stage in GSE39582.


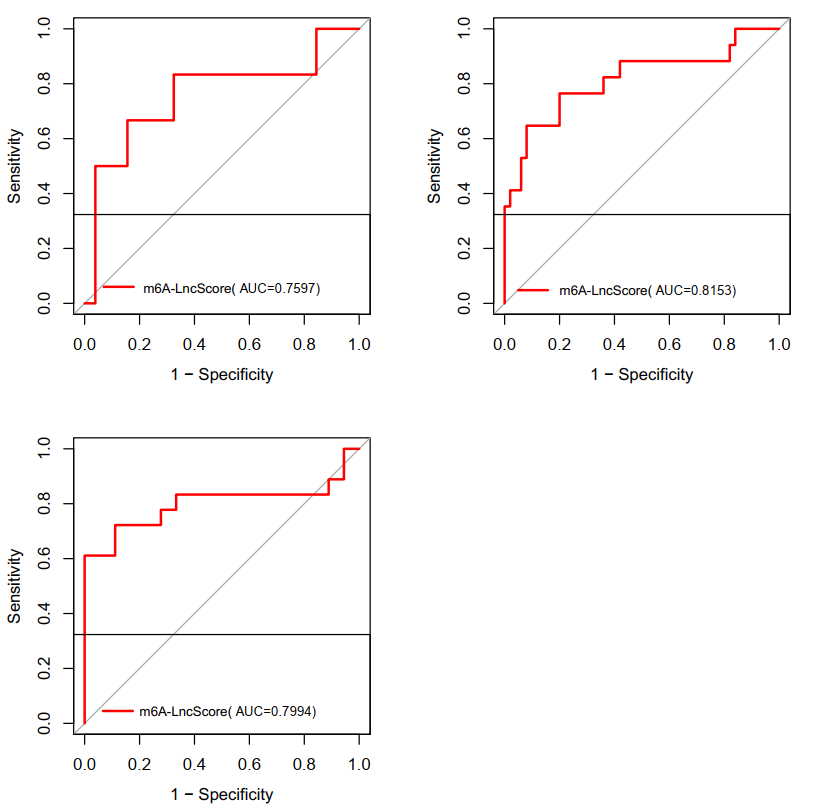


**Figure S10** The ROC curve plot of m6A-LncScore for predicting PFS in GSE33113. Since univariate regression analysis had no significant features except m6A-LncScore, ROC analysis was performed only for m6A-LncScore.


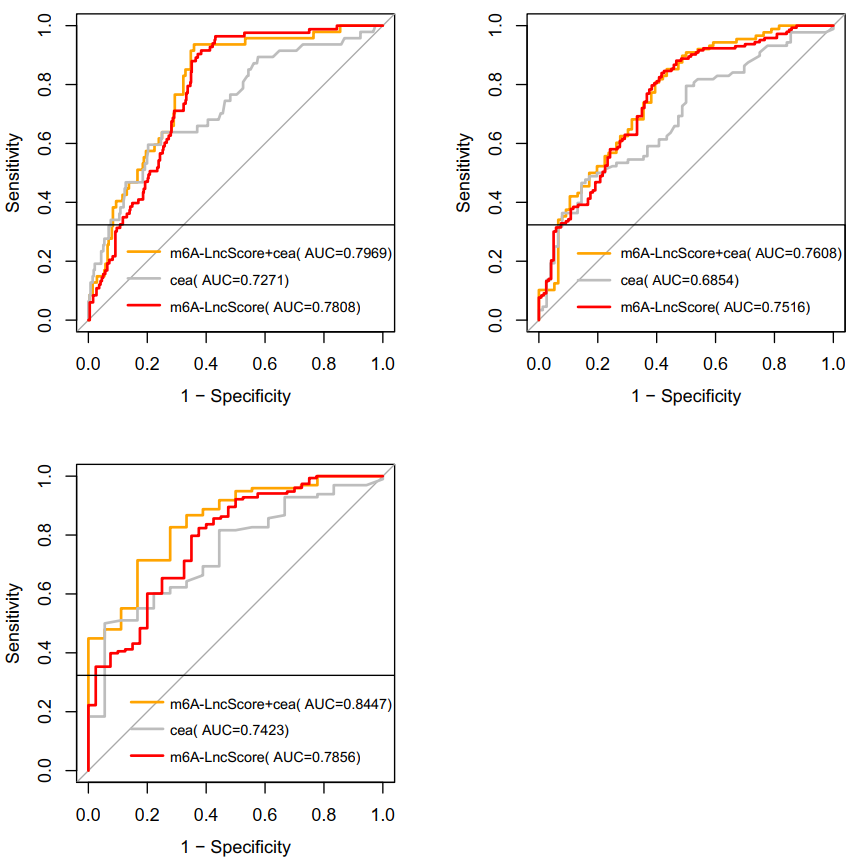


**Figure S11** Integrated model for predicting PFS was [superior](D:/Program%20Files%20(x86)/Youdao/Dict/8.9.4.0/resultui/html/index.html#/javascript:;) [to](D:/Program%20Files%20(x86)/Youdao/Dict/8.9.4.0/resultui/html/index.html#/javascript:;) m6A-LncScore or CEA level.


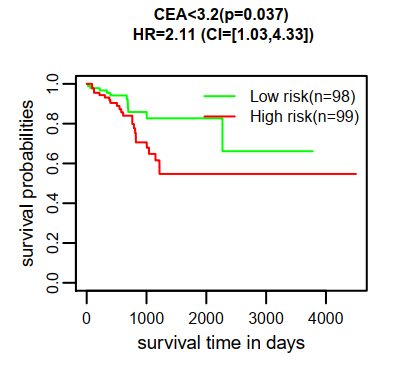

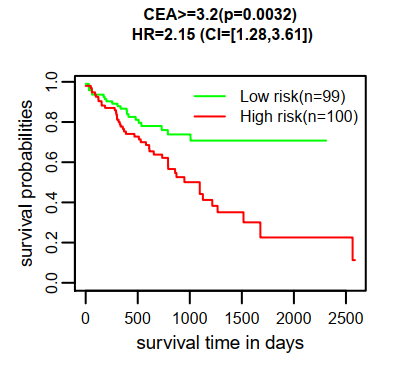


**Figure S12** Stratification analysis shows m6A-LncScore is not dependent on CEA level for predicting PFS.


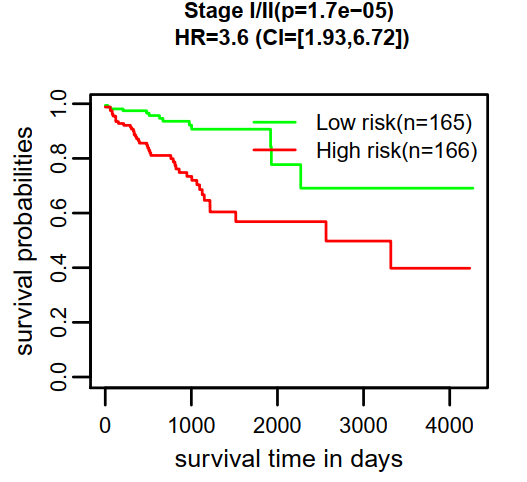

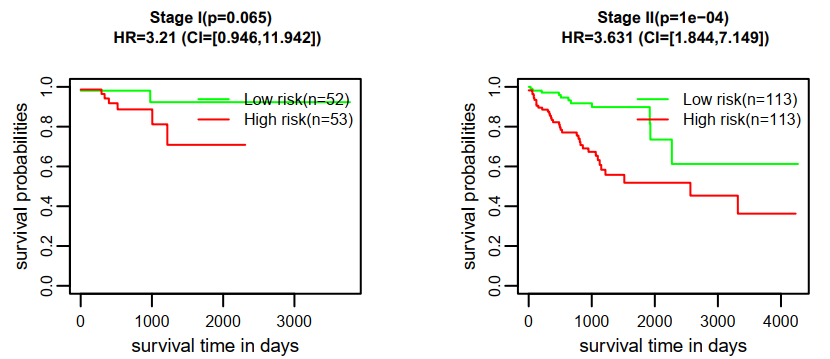


**Figure S13** m6A-LncScore is beneficial to stratify early cancer stages patients into risk groups, especially in stage II patients.

**Legends of Supplementary tables**

**Table S1** The basic clinical information of patients from our center.

Attached

**Table S2** The Primers used in this study.

Attached

**Table S3** The differential expression information of 43 lncRNAs in TCGA dataset.

Attached **Table S4** 18 lncRNAs interact with m6A regulators from NPInter V4.

Attached

**Table S5** 24 m6A-related lncRNAs could be methylated or demethylated by m6A writers or erasers in M6A2TARGET.

Attached

**Table S6** 24 m6A-related lncRNAs could bind to m6A readers in M6A2TARGET.

Attached

**Table S7** The expression of 24 m6A-related lncRNAs could be influenced by over-expression or knock down of m6A regulators in M6A2TARGET.

Attached

**Table S8** 24 m6A-related lncRNAs are significantly co-expressed with at least one m6A regulator in TCGA dataset.

Attached

**Table S9** The regression coefficients in LASSO analysis.

Attached

**Table S10** The expression of 5 lncRNAs in 55 pairs of samples detected by qRT-PCR.

Attached

**Table S11** The univariate Cox regression analysis result of m6A-LncScore and clinicopathologic features in three datasets.

Attached

**Table S12** The multi-variate Cox regression analysis result of m6A-LncScore and clinicopathologic features in three datasets.

Attached

**Table S13** The comparison of integrated model with m6A-LncScore and tumor stage.

Attached

**Table S14** The comparison of integrated model with m6A-LncScore and tumor stage in three additional datasets.

Attached

**Table S15** The comparison of integrated model with m6A-LncScore and CEA level in TCGA dataset.

Attached

**Table S16** The comparison of m6A-LncSig and three known lncRNA signatures.

Attached
